# Modulation of the optical transmittance in monolayer graphene oxide by using external electric field

**DOI:** 10.1038/srep14441

**Published:** 2015-09-25

**Authors:** Zhixing Qiao, Chengbing Qin, Yan Gao, Guofeng Zhang, Ruiyun Chen, Liantuan Xiao, Suotang Jia

**Affiliations:** 1 State Key Laboratory of Quantum Optics and Quantum Optics Devices, Institute of Laser Spectroscopy, Shanxi University, Taiyuan 030006, China

## Abstract

Graphene oxide (GO) emerges as a functional material in optoelectronic devices due to its broad spectrum response and abundant optical properties. In this article, it is demonstrated that the change of optical transmittance amplitude for monolayer GO (mGO) could be up to 24.8% by an external electric field. The frequency harmonics for transmittance spectra are analyzed by use of Fast Fourier Transforms to give an insight into the modulation mechanism. Two physical models, the electrical permittivity and the sheet conductivity which linearly vary as the electric field, are proposed to response for the transmittance modulation. The model-based simulations agree reasonable well with the experimental results.

Graphene oxide (GO), as one of the most important derivatives of graphene, is a layered material featuring a variety of oxygen-containing functionalities with epoxy and hydroxyl groups on the basal plane and carbonyl and carboxyl groups along the edges^1^,^2^. The GO’s chemically inhomogeneous and spatially disordered structures result in abundant optical properties, which have been extensively studied both experimentally^3^,^4^,^5^ and theoretically^6^,^7^, respectively. The GO’s unique optical properties have been used to design devices and sensors, such as electro-optic modulators^8^, fluorescence-sensors^9^, and laser absorption medium^10^ *et al.*

There are many attempts, such as chemical and photocatalytic reduction^11^,^12^, thermal annealing procedure^13^, and solvent effect^14^, have been used to improve and control GO’s optical properties. As one of the most effective and environmentally friendly methods, electric field has been used to modulate the GO’s optical properties. In 2011, Ekiz *et al.*^15^ observed the reversible reduction and oxidation of multi-layers GO films under the electrical stimulus, resulting in switching between partially reduced graphene oxide and graphene. Later Ciraci *et al.*^16^ investigated the effects of the electric field on the oxidation/deoxidation of GO as absorption/desorption of oxygen atoms from epoxy groups by first-principles calculations. In 2012, Hu *et al.*^17^ studied the electrically controlled electron transfer of thionine-functionalized reduced GO in the form of films. Hu’s schemes could be used to fabricate the resistance random access memories which showed nonvolatile resistive switching with large ON/OFF ratio. However, the modulations of the optical transmittance in monolayer GO (mGO) by external electric field with sub-micrometer resolution have not been reported.

In this work, the experimental observations of the modulation of optical transmittance in mGO under external electric field by scanning confocal microscope will be demonstrated, with the spatial resolution on GO basal plane about ~300 nm determined by the optical diffraction limit. The various modulated transmittance results, arising from GO’s chemically inhomogeneous and spatially disordered structures, will be presented. Based on the electrical permittivity and sheet conductivity of mGO, which are linear dependent on the external electric field, the theoretical analysis and simulations with deduced parameters as well as the comparison with the experimental results will be shown.

## Results

### Device structure, AFM, Raman and XRD spectra

**Figure 1:**
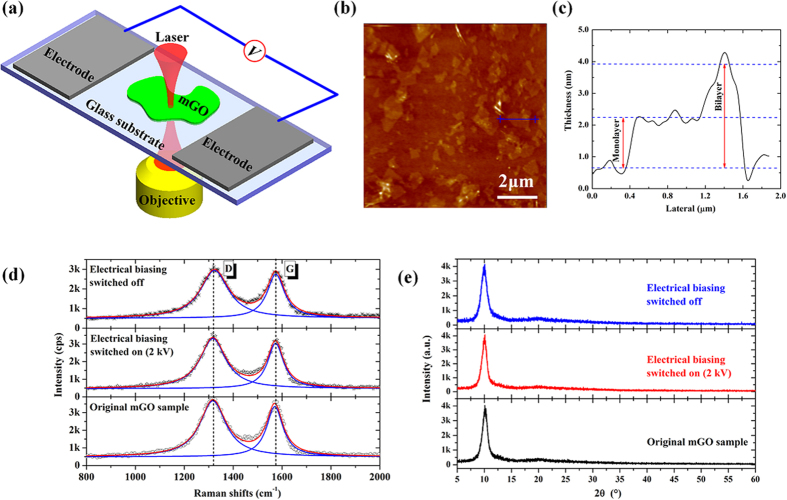
(**a**) Schematic diagram of the electrode device structure. (**b**,**c**) are AFM image of mGO sample spin coated on the glass substrate and the heights data for the selected parts in the straight line. (**d**,**e**) are Raman and XRD spectra for the original mGO sample, as well as the mGO with electrical biasing switched on and off, respectively.

Figure 1(a) shows the schematic diagram of electrode device structure. The GO material (purity > 99%, the single layer ratio ~99%, and the diameter about 1 ~ 5 μm) was commercially available water dispersion, purchasing from Nanjing XFNANO Materials Tech Co. Ltd. After diluted with deionized water, 100 μL GO dispersion with concentration of 5 × 10^−3^ mg/ml was spin-coated onto a glass substrate. The as-prepared GO sample was dried at room temperature in vacuum conditions for 24 hours to removal the residual water. A pair of aluminum electrodes was fabricated on the glass substrate with the space of 2 mm. A sine wave electrical biasing applied onto the GO sample was generated from a function generator (Agilent, 33250) and amplified to ±2 kV by a high voltage model (Tjshenghuo Tech CO., HVA-502R) before connecting to the electrodes. The laser beam was focused by an objective (Nikon, NA = 1.3, 100×) to the sample plane with the diffraction spot about 300 nm. All the experiments were performed under atmosphere condition at room temperature with the relative humidity about 8%.

The atomic force microscopy (AFM) image of the GO sheet is illustrated in Fig. 1(b). It is determined that the thickness of the resulting GO sample was ~1.5 nm, corresponding to the structure characterization of mGO^18^, as shown in Fig. 1(c). Partial overlapping GO films exhibited a thickness of ~3.0 nm, corresponding to the bilayer GO. The typical Raman spectra for the original mGO sample, as well as the mGO with electrical biasing switched on and off are presented in Fig. 1(d), respectively. The spectra were fitted by Lorentz function with two components of D and G bands. Neither the Raman shifts for D and G bands nor their intensity ratios (I_D_/I_G_), which could indicate the disordered degree of graphitized structure that contains oxygen-containing functional groups and defects^19^,^20^, have significant change under these three conditions. The results revealed that no obvious oxidation and/or reduction occurred to mGO sample when electrical biasing applied. Figure 1(e) presents the X-ray diffraction (XRD) spectra under three conditions as that in Fig. 1(d), where the main diffraction peaks and the full widths at half maximum (FWHM) are similar. The XRD results further suggested that there is no reduction during electrical biasing. The detailed fitting results for Raman and XRD spectra are presented in supporting information.

### Transmittance modulated by external electric field

**Figure 2:**
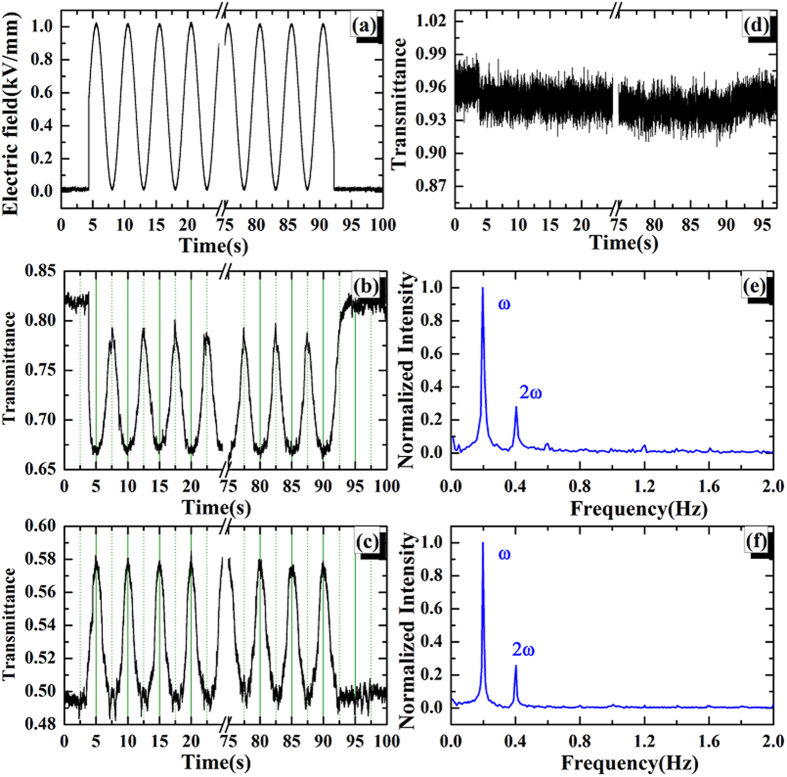
(**a**) The unipolar sine wave electric field with a form of *E *= *E*_*0*_(sin(*ωt*) + 1) (*E *= 0.5 kV/mm, *ω *= 0.2 Hz). The cycles with 5 s have been indicated by dash lines. (**b–d**) are the modulated transmittance trajectories for selected mGO spots varied as the electric field *E*, respectively. (**e,f**) are the corresponding FFTs (normalized) for the transmittance of (**b,c**), respectively. The first (*ω*) and second (2*ω*) harmonics have been labeled in frequency domain.

The optical transmittance of mGO varied as the electric field has been obtained by keeping the laser focusing on the selected mGO spots and applying the alternating high voltages on the electrodes. Typical transmittance trajectories varied as a function of electric field are presented in Fig. 2. Here the voltage is unipolar (i.e. positive) in the form of *V *= *V*_*0*_(sin(*ωt*)+1) with *V*_*0 *_= 1 kV, and *ω *= 0.2 Hz, respectively. With 2 mm space between two electrodes, the electric field in the region of 0–1 kV/mm (Fig. 2(a)) is generated. As shown in Fig. 2(b), the transmittance for some mGO spots are gradually decreased as the electric field rising, while for others the transmittance might be increased, as shown in Fig. 2(c). There are also some spots whose transmittance exhibit weak response to the external electric field, as given in Fig. 2(d). A common result for all selected spots is that the transmittance can be recovered when the electric field is turned off. In order to quantitatively describe the transmittance variation, here we define the change in optical transmission amplitude as *C*_T _= |(*T*_mod_–*T*_ini_)/*T*_ini_)| × 100%, where *T*_mod_ and *T*_ini_ are the maximum or minimum modulated transmittance and initial transmittance, respectively. The *C*_T_ for Fig. 2(b,c) are 18.5% and 16.6%, respectively, which is significantly larger than that in graphene (1% ~ 5%)^21^. In order to get deep insight into the modulation phenomenon, we perform the numerical Fast Fourier Transforms (FFTs) in frequency domain, as presented in Fig. 2(e,f), respectively. Despite the first harmonic (*ω*) dominates the frequency, significant second harmonic (2*ω*) signal can be observed, which indicates the nonlinear response of the mGO’s transmittance to the external electric field.

**Figure 3:**
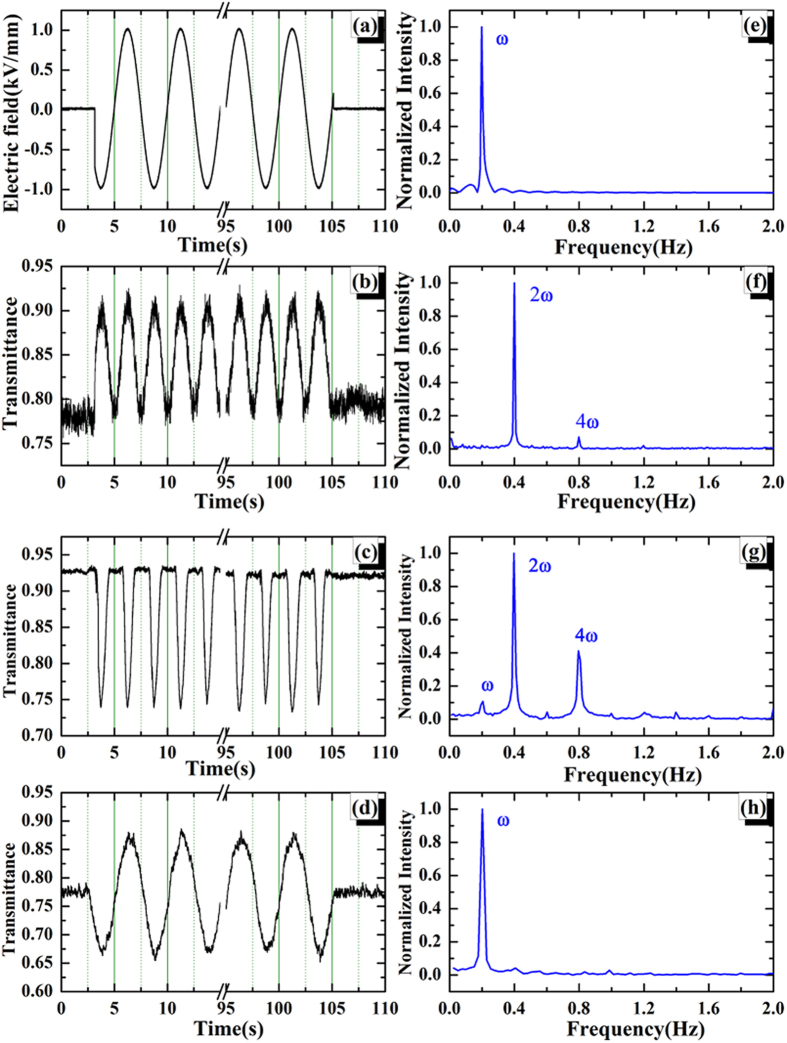
(**a**) The bipolar sine wave electric field with a form of *E *= *E*_*0*_sin(*ωt*) (*E *= 1 kV/mm, *ω *= 0.2 Hz ). The cycles with 5 s have been indicated by dash lines. (**b**–**d**) are the modulated transmittance trajectories for selected mGO spots varied as the electric field *E*, respectively. (**e**) is the FFT for the sine wave in (**a**). (**f**–**h**) are the corresponding FFTs (normalized) for the transmittance of (**b**–**d**), respectively. The main harmonic signals have been labeled in frequency domain.

Beyond the unipolar electric field, the bipolar electric field (i.e. positive and negative alternately) has also been performed to the mGO sample, where the voltage is in the form of *V *= *V*_*0*_sin(*ωt*) (Fig. 3(a)), with *V*_*0 *_= 2 kV and *ω *= 0.2 Hz, respectively. For some spots, the transmittance are decreased or increased under both positive and negative electric field, as shown in Fig. 3(b,c), respectively. In this case, the second harmonic frequency (2*ω*) is the major component in FFT, as presented in Fig. 3(f,g), respectively. Besides, there also exist the modulated results that the transmittance is increased in positive field and decreased in negative field, as presented in Fig. 3(d). In this situation, the first harmonic signal dominates over all the others (Fig. 3(h)).

**Figure 4:**
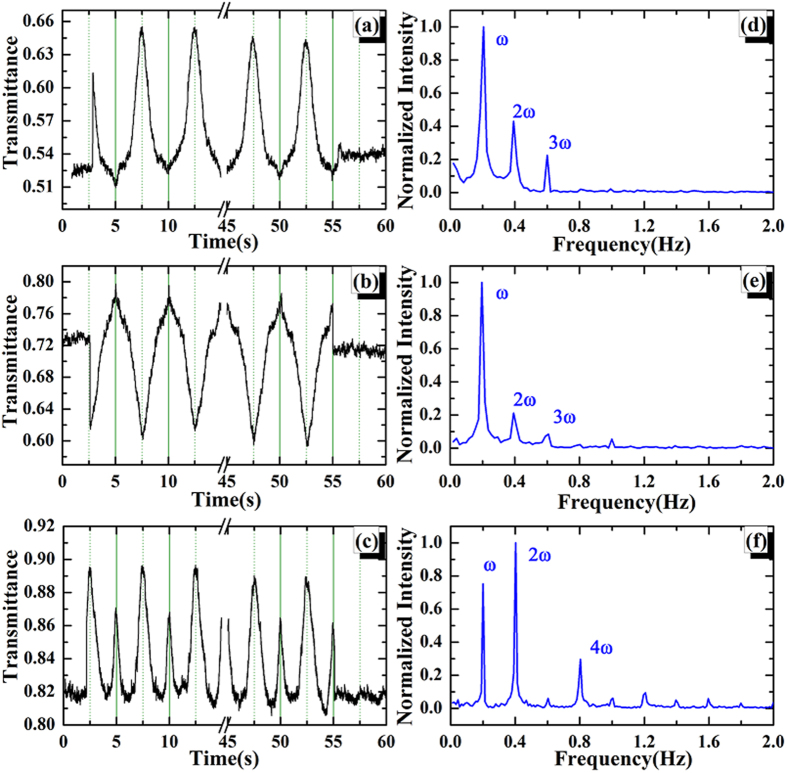
(**a**–**c**) are the modulated transmittance trajectories for selected mGO spots varied as the electric field *E *= *E*_*0*_sin(*ωt*), respectively. The cycles with 5 s have been indicated by dash lines. (**d**–**f**) are the corresponding FFTs (normalized) for the transmittance of (**a**–**c**), respectively. The main harmonic signals have been labeled in frequency domain.

Excepting the symmetric transmittance profiles in bipolar electric field, there exist some asymmetric results, as shown in Fig. 4(a,b). It can be found that when the *C*_T_ in onefold field (either positive or negative) is large enough, no obvious modulation can be observed in the opposite direction. In Fig. 4(a), the *C*_T_ in negative field is 21%, while it’s just about 2% in positive field. In this case, the first, second and third harmonics play important roles in modulated transmittance, as shown in Fig. 4(d,e), respectively. In addition, Fig. 4(c) presents the modulated transmittance with asymmetric *C*_T_ in positive and negative electric fields, which shows many harmonics in frequency domain, as given in Fig. 4(f). This result is fully agreed with the electric field modulated transmittance in graphene, as shown in ref. 21. The comparison of *C*_T_ for the selected spots mentioned above is shown in Table 1.

Here we propose two possible mechanisms to explain the transmittance modulated by electric field. One is mGO’s electrical permittivity varies as a function of the external electric field, resulting from the nonlinear polarization. Another is that the transmittance depends on the mGO’s sheet conductivity, originating from the electric-field-driven tunneling effects.

### Electrical permittivity varies as the electric field

The high order harmonics in frequency domain, as shown in FFT results, could be understood as the nonlinear polarization of mGO to the external electric field, which can be expressed as:


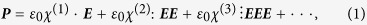


where ***P*** and ***E*** are the polarization and external electric field, *χ*^(*i*)^ (*i *> 1) are the *i*th order nonlinear susceptibilities, *ε*_0_ is the vacuum permittivity. Here, we just consider the first two terms in the right part of equation (1). As a first approximation, we suppose that the mGO’s susceptibilities is linear dependent on the external electric field *E* in the units of kV/mm, in the form of 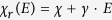, where *χ* is the constant susceptibility for all fields, and 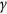 is a proportionality which will be held for the selected spot. In this case, the polarization can be given just by


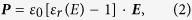


where


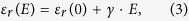


and 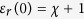, based on the fact that 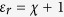.

On the other hand, the transmittance *T* of mGO’s with thickness *d* can be calculated by using the Lambert-Beer law, 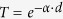. The absorption coefficient, α, can be given by 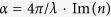, where Im(*n*) is the imaginary part of the refractive index, and *λ* is the wavelength of the transmitted light. According to the Maxwell equations, Im(*n*) can be expressed in terms of the real and imaginary part of the electric permittivity, Re(*ε*_*r*_) and Im(*ε*_*r*_), as follows:


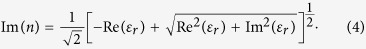


Hence, the optical transmittance *T* can be expressed as:


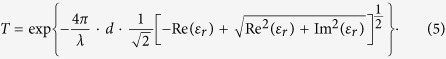


When Im(*ε*_*r*_) is a constant^21^, the response for electrical permittivity (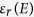) to electric field is only dependent on Re(*ε*_*r*_). Therefore, the transmittance will vary as the function of Re(*ε*_*r*_). Once the 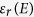 in equation (3) is determined, the transmittance varies as the alternating electric field can be obtained.

**Figure 5:**
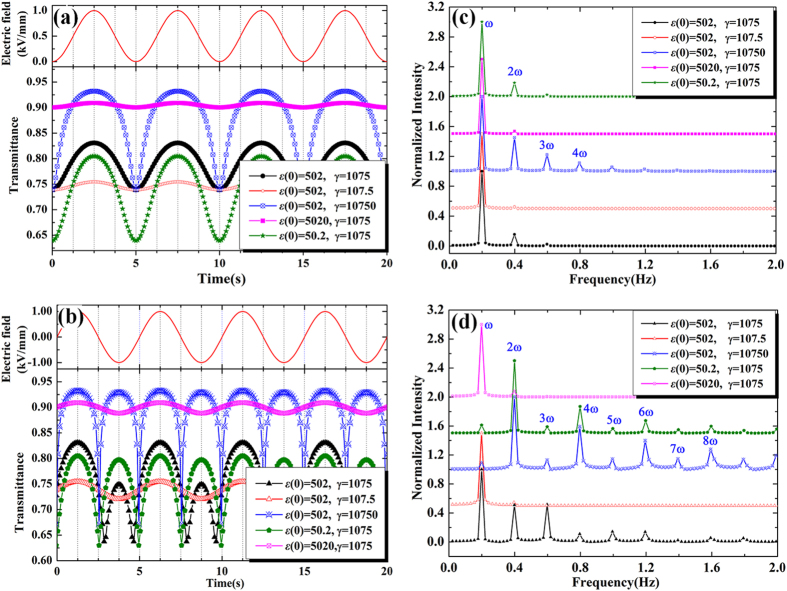
(**a**,**c**) are the simulations calculated from equation (5) under the electric field wave of *E *= *E*_*0*_(sin(*ωt*) *+ *1) (*E *= 0.5 kV/mm) and the corresponding FFTs, respectively. (**b**,**d**) are the simulations calculated from equation (5) under the electric field wave of *E *= *E*_*0*_sin(*ωt*) (*E *= 1.0 kV/mm) and the corresponding FFTs, respectively. The main harmonic signals have been labeled in frequency domain.

The 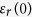 and 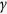 can be deduced from the experimental data of Ref. 22which investigated the ferroelectricity of GO^22^, giving 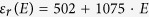, with 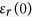 = 502 and 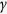 = 1075 mm/kV. In our experimental condition, λ = 635 nm and *d *= 1.5 nm. Taking *E *= 0.5(sin(*ωt*) + 1) or *E *= sin(*ωt*), and substituting equation (3) to equation (5), the calculated transmittance are presented in Fig. 5(a,b), respectively. Both results show that the transmittance is increased when the field rises. Due to the anisotropy of the selected spots, the 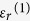 and 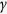 might be quite different, therefore the simulations with varied parameters and their corresponding FFTs in frequency domain are also presented in Fig. 5(a–d), respectively. The simulations and FFTs agree reasonable well with the experimental results. The simulated parameters for the selected spots are presented in Table 1.

### Sheet conductivity varies as the electric field

**Figure 6:**
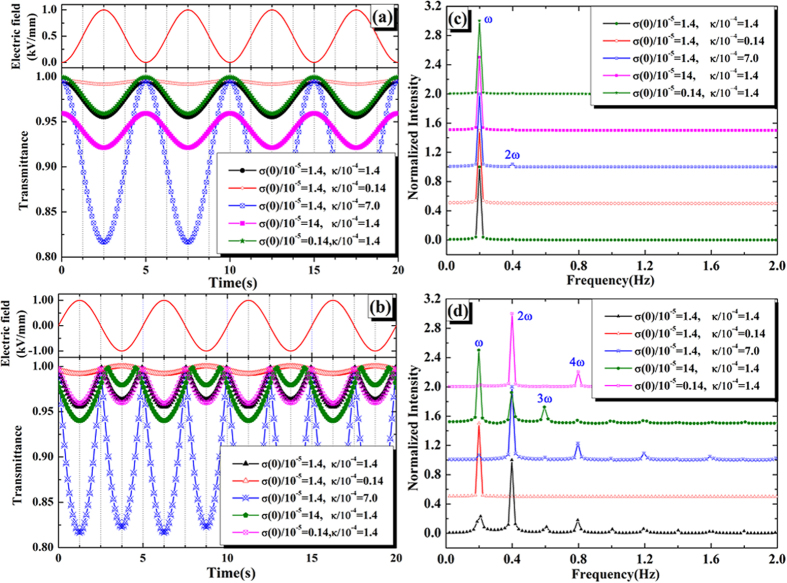
(**a,c**) are the simulations calculated from equation (8) under the electric field wave of *E *= *E*_*0*_(sin(*ωt*) + 1) (*E *= 0.5 kV/mm) and the corresponding FFTs, respectively. (**b**,**d**) are the simulations calculated from equation (8) under the electric field wave of *E *= *E*_*0*_sin(*ωt*) (*E *= 1.0 kV/mm) and the corresponding FFTs, respectively. The main harmonic signals have been labeled in frequency domain.

The second possible mechanism is based on the observation of electric-field-driven tunneling in oxidative functionalization of monolayer graphene and chemically derived graphene monolayers^23^. In the case, the sheet conductivity of mGO will be tuned by the external electric field (See supporting information, Figure S3). It has been pointed that the conductivity of the individual mGO sheets depended on the magnitude of external electric field and temperature^24^. On the other hand, the transmittance for glass-GO-air structure is rely on the sheet conductivity and can be modeled as


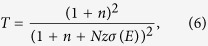


which has been used for the case of multilayer graphene^25^. This equation is deduced by using thin-film Fresnel coefficients and the Drude model^26^, where mGO is considered as a zero-thickness conductive film, and glass substrate is treated as an optical thick dielectric medium. Here *n = *1.523 is the refractive index of the glass substrate, *N *= 1 is the number of GO’s layers, *z *≈ 376.73 Ω is the free-space impedance, and σ(*E*) denotes the sheet conductivity of mGO which varies as a function of electric field. Here we suppose the conductivity is linear dependent on the electric field *E* in the units of kV/mm, in the form of


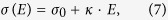


in which σ_0_ is a constant, and *κ* is a proportionality which will be held for the selected spot. By using the date from ref. 27, the value of σ(*E*) can be deduced as


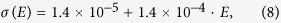


where σ_0_/10^−5^ = 1.4, and *κ*/10^−4^ = 1.4. Taking *E *=  0.5(sin(*ωt*)+1) or *E *= sin(*ωt*), and substituting equation (8) to equation (6), the calculated transmittance are presented in Fig. 6(a,b), respectively. Considering the anisotropy of the selected spots, the simulations with other parameters are calculated. Figure 6(c,d) present their FFTs results. Both the simulations and FFTs are in agreement with experimental data well.

From the above two physical mechanisms, it can be found that the modulated transmittance is increased as the electric field rises in the case of electrical permittivity mechanism (Fig. 5), while it is decreased when considering the sheet conductivity mechanism (Fig. 6). In other words, these two mechanisms lead to different modulated results, which could be used to explore the varied experimental data. The result shown in Fig. 3(b) would originate from the change of electrical permittivity, nevertheless, the result shown in Fig. 3(c) might root from the vary of sheet conductivity. Therefore, the transmittance modulated by electric field might originate from each of the two mechanisms.

Despite the conductivity of GO is also temperature dependent^24^, the *C*_T_ derived from the temperate effect is extremely small (*C*_T_ is about 2 × 10^−8^ when the temperature varies from 310 to 350 K). The change in optical transmittance resulting from the varied temperature should be much less than 1% in the experiment. Thus, temperature effect cannot attribute to our significantly modulated results (~20%).

## Discussion

The modulation of the optical transmittance of mGO has been achieved by sine wave electric field, and the anisotropic optical properties in one block GO are also presented. Except the fundamental modulated frequency, the higher order harmonics are determined by FFT with the nonlinear relationship between GO’s polarization and external electric field. The electrical permittivity and sheet conductivity of mGO, which are connected with the transmittance, are used to explain the modulated transmittance. The simulations with deduced parameters agree reasonable well with the experimental results. The transmittance controlled by electric field has potential applications in the fabrication of GO-based electro-optical and electrochromic devices. This phenomenon also opens the generation of electrical signals with higher frequencies than the excitation, and performs the possibility of using this control in the transmission of information by optical means^21^.

## Methods

### Transmittance measurements

The transmitted spectrum was recorded with a home-built scanning confocal microscope^28^,^29^ based on an invert microscope (Nikon, TE2000-U). The schematic diagram of the experimental apparatus was shown in Figure S1. A cw 635-nm diode laser (PicoQuant, LDH-D-C-635) was used to excite the GO sample. After intensity modulated by an acoustic optical modulator (AOM, Crystal Technology, 3080-122) with a 50 kHz sine wave, the laser beam was split into two optical paths by a glass with the ratio about 5:95. The weaker beam was used as a reference to monitor the fluctuation of the laser power by a Si photodiode (PD1, Femto, HSA-X-S-1GB-SI-FS), while the stronger beam was sent through a beam expander (BE) and an excitation filter (ExF, Semrock). And then the laser was directed to a dichroic mirror (DM, Semrock, Cy5-4040A) towards an oil objective (OBJ, Nikon, NA = 1.3, 100×) to obtain a diffraction limited spot (~300 nm) on the sample plane. After passing through the GO sample, the laser was focused into another Si photodiode (PD2, FEMTO, HCA-S-400A). The signals from PD1 and PD2 were both demodulated by lock-in amplifiers (Stanford Research, SR830) and then digitized by a data acquisition (DAQ, NI6251) card interfaced to a personal computer (PC) to give the transmission intensity. The mGO’s transmittance is determined by comparing the transmission intensity of mGO sample with that of glass substrate (the background).

### Raman, AFM and XRD characterization

The Raman spectrum of mGO sample was collected by the same objective and went across DM again. After passing through a notch filter (NF, Semrock, NF03-633E-25), the Raman scattering was transmitted through the optical fiber and measured by a monochromator (Horiba Jobin Yvon, 1250M). AFM image was taken by CSPM5500 scanning probe microscope (Being Nano-Instruments, Ltd.). X-ray diffraction measurements were carried out by use of a Rigaku BD2000 system with scanning rate of 4°min^−1^ from 5° to 60° (Cu Kα X-rays of 0.154 nm) operating at 30 kV and 20 mA. Here we are grateful to Professor Xiaotian Qu for XRD measurements.

## Additional Information

**How to cite this article**: Qiao, Z. *et al.* Modulation of the optical transmittance in monolayer graphene oxide by using external electric field. *Sci. Rep.* **5**, 14441; doi: 10.1038/srep14441 (2015).

## Supplementary Material

Supplementary Information

## Figures and Tables

**Table 1:**
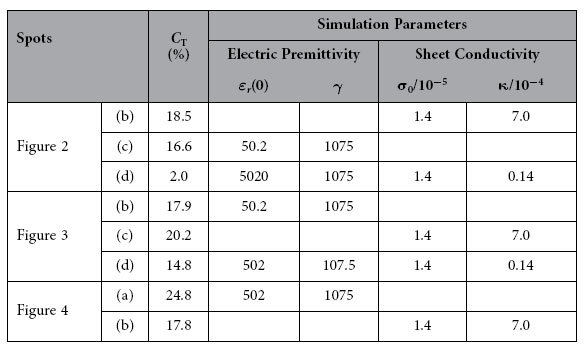
The change in optical transmission amplitude (*C*_T_) and the appropriate simulated parameters for the selected spots in the manuscript.
