# Supplementary material for: Modulation of the optical transmittance in monolayer graphene oxide by using external electric field
Source: Sci Rep. 2015 Sep 25;5:14441. doi: 10.1038/srep14441 (PMC5155675; doi:10.1038/srep14441)
Supplement: Supplementary Information [file srep14441-s1.pdf]

Supplementary information

Modulation of the optical transmittance in monolayer graphene oxide by using external electric field

Zhixing Qiao, Chengbing Qin*, Yan Gao, Guofeng Zhang, Ruiyun Chen, Liantuan Xiao* and Suotang Jia

State Key Laboratory of Quantum Optics and Quantum Optics Devices, Institute of Laser Spectroscopy, Shanxi University, Taiyuan 030006, China

*Corresponding author: chbqin@sxu.edu.cn & xlt@sxu.edu.cn

Content

1. Schematic diagram of the experimental apparatus;

2. Fitting results for Raman spectra;

3. Fitting results for X-ray diffraction (XRD) spectra;

4. The sheet conductivity measurements for GO films.

1. Schematic diagram of the experimental apparatus;

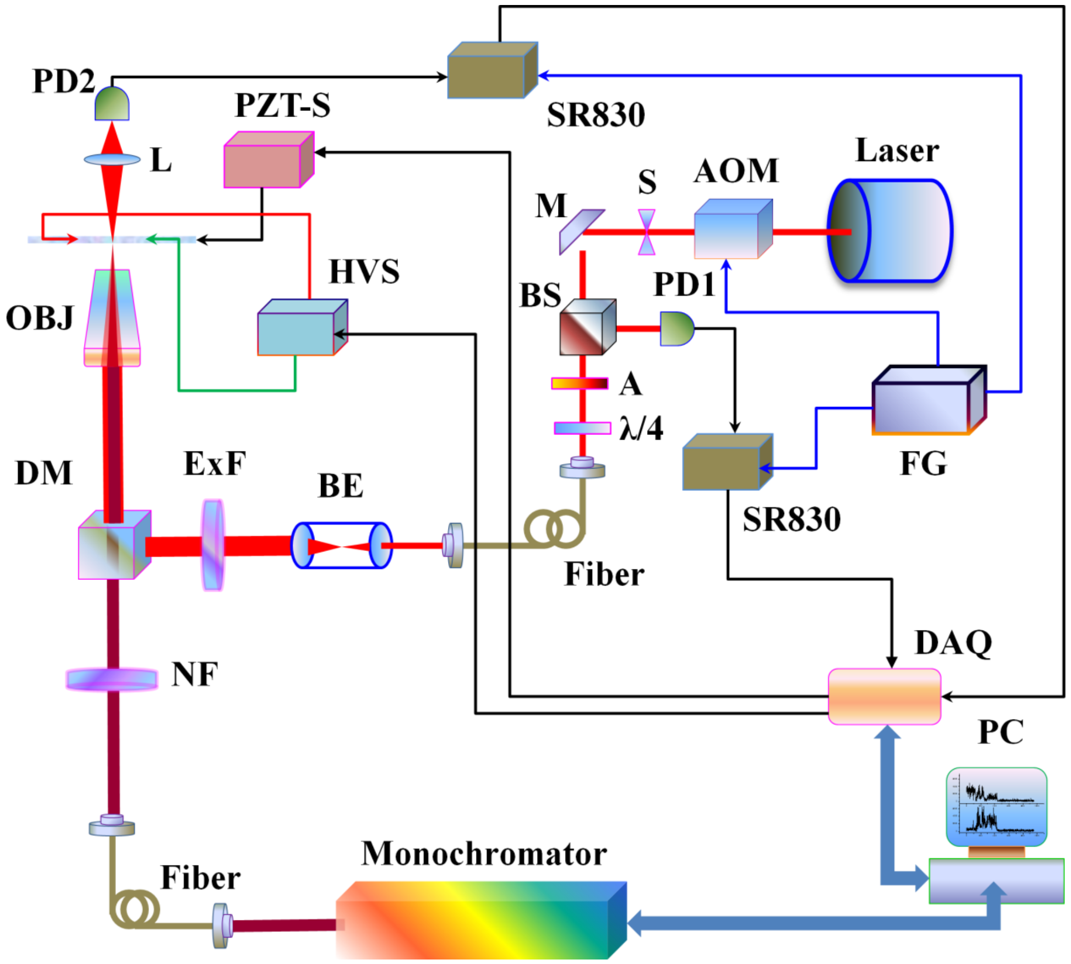

Figure S1. Schematic diagram of the experimental apparatus.

AOM: acoustic optical modulator, S: shutter, M: mirror, G: glass, PD: Si photodiode, A: attenuator; BE: beam expander, ExF: excitation filter, DM: dichroic mirror, OBJ: objective, L: lens, HVA: high voltage amplifier, PZT: piezoelectric-ceramics, SR830: lock-in amplifier, FG: function generator, DAQ: data acquisition card, NF: notch filter, and PC: personal computer.

2. Fitting results for Raman spectra;

The mGO's Raman spectra for the original mGO sample, as well as the mGO with electrical biasing switched on and off, as shown in Figure 1(d), were fitted by Lorentz function with two components: D and G bands. Here the determined Raman shifts(ω), full width at half maximum (FWHM, Δ), fluorescence intensity (I) for D and G bands, as well as their intensity ratios (I_D/I_G), respectively, are presented in Table S1. Even though the FWHMs for D and G bands with the electrical biasing switched on (2kV) are a little broader than that for original mGO sample, and the Raman shifts are a slight red shift, the Raman shifts and FWHMs for mGO with the electrical biasing switched off are almost identical to the original mGO sample. Furthermore, the intensity ratios (I_D/I_G), which could indicate the disordered degree of graphitized structure that contains oxygen-containing functional groups and defects, are also similar before and after electrical biasing. The results proved that there are no permanent reduction of done during the electrical biasing applied.

Table S1 The comparison of Raman shift (ω), full width at half maximum (Δ), fluorescence intensity (I) and ratio of D band and G band (I_D/I_G).

Type	Band	ω/cm^{-1}	Δ/cm^{-1}	I	I_D/I_G
Original	D	1316.9(7)	128(2)	3179	1.11
	G	1570.0(6)	81(2)	2856	
Electrical Biasing switched on (2 kV)	D	1323.4(8)	136(3)	2431	1.08
	G	1574.2(6)	85(2)	2244	
Electrical Biasing switched off	D	1316.9(7)	127(2)	2850	1.10
	G	1571.3(4)	79(2)	2578	

3. Fitting results for X-ray diffraction (XRD) spectra;

The mian diffraction peaks (~10 °) of mGO's XRD spectra for the original mGO sample, as well as the mGO with electrical biasing switched on and off were fitted by Lorentz function, as shown in Figure S2. It can be found that neither peak positions and FWHM nor their intensity have significant change. The results further proved that there are no permanent reduction of done during the electrical biasing applied.

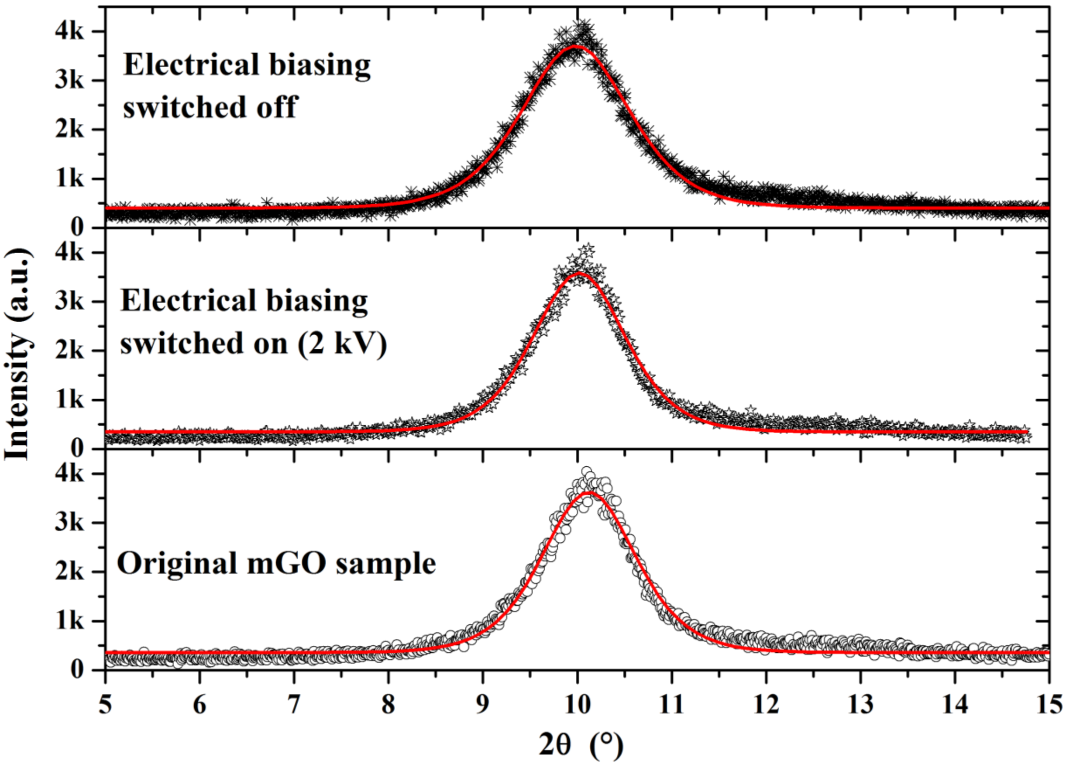

Figure S2 The mGO's XRD spectra for the original mGO sample, as well as the mGO with electrical biasing switched on and off, and their fitting results, respectively.

4. The sheet conductivity measurements for GO films.

In order to determine the sheet conductivity varied as electric field, we performed the *I-V* measurement for the GO films at room temperature with the relative humidity of 8%. The result is shown in Figure S3. It can be found that the current is extremely low in the voltage range of -1.0 kV to 1.0 kV. Naturally, the sheet conductivity has no obvious change in this region, corresponding to no obvious response to the electric field, as shown in Figure 3(c). When the voltage bigger that 1.0kV, the current is not linear varied as the voltage, which indicants the sheet conductivity is changed with applied voltage, resulting in transmittance altered with electric field, as shown in manuscript.

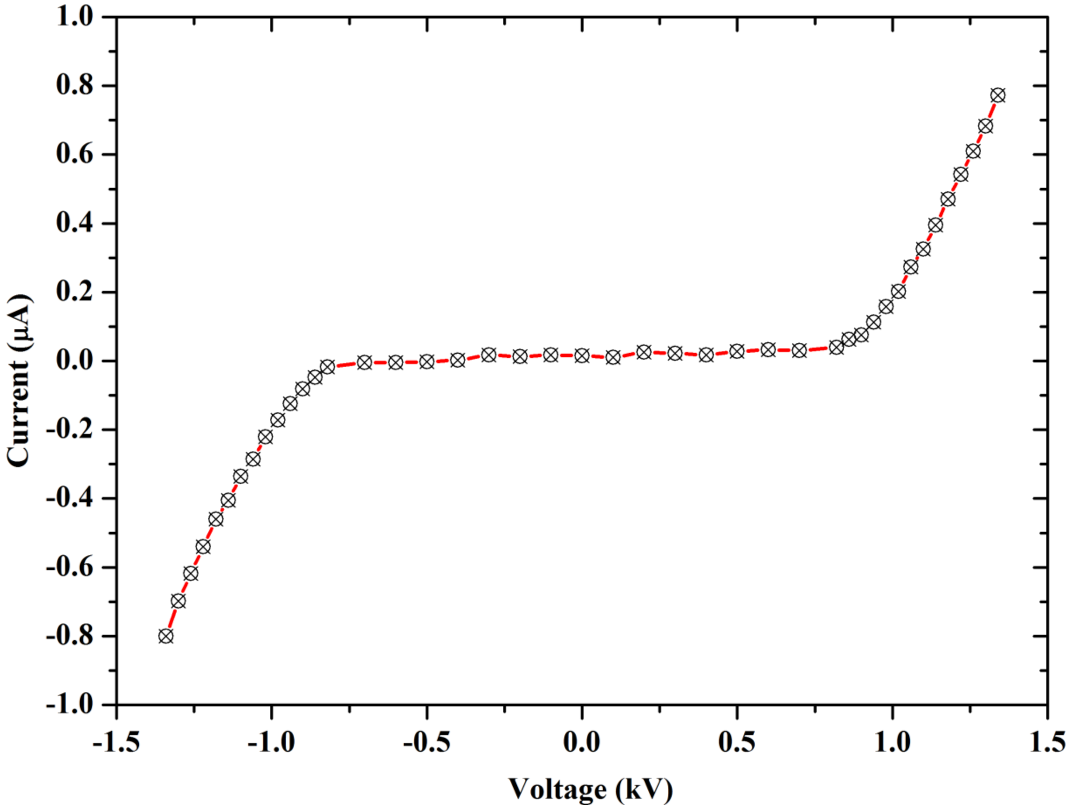

Figure S3 Measured I-V characteristic of GO films applied with sweeping voltage -2.0 to 2.0 kV in the relative humidity of 8% at room temperature.
